# Widespread Exonization of Transposable Elements in Human Coding Sequences is Associated with Epigenetic Regulation of Transcription

**DOI:** 10.4172/2329-8936.1000101

**Published:** 2013-06-19

**Authors:** Ahsan Huda, Pierre R Bushel

**Affiliations:** 1 Microarray and Genome Informatics Group, National Institute of Environmental Health Sciences, USA; 2 Kelly Government Solutions, Inc., USA; 3 Biostatistics Branch, National Institute of Environmental Health Sciences, Research Triangle Park, NC 27709, USA

**Keywords:** Gene expression, Transcription, Transposable elements, Exonization, Epigenetics, RNA-seq, DNAseI HS, Alu, LTR Retrotransposons

## Abstract

**Background:**

Transposable Elements (TEs) have long been regarded as selfish or junk DNA having little or no role in the regulation or functioning of the human genome. However, over the past several years this view came to be challenged as several studies provided anecdotal as well as global evidence for the contribution of TEs to the regulatory and coding needs of human genes. In this study, we explored the incorporation and epigenetic regulation of coding sequences donated by TEs using gene expression and other ancillary genomics data from two human hematopoietic cell-lines: GM12878 (a lymphoblastoid cell line) and K562 (a Chronic Myelogenous Leukemia cell line). In each cell line, we found several thousand instances of TEs donating coding sequences to human genes. We compared the transcriptome assembly of the RNA sequencing (RNA-Seq) reads with and without the aid of a reference transcriptome and found that the percentage of genes that incorporate TEs in their coding sequences is significantly greater than that obtained from the reference transcriptome assemblies using Refseq and Gencode gene models. We also used histone modifications chromatin immunoprecipitation sequencing (ChIP-Seq) data, Cap Analysis of Gene Expression (CAGE) data and DNAseI Hypersensitivity Site (DHS) data to demonstrate the epigenetic regulation of the TE derived coding sequences. Our results suggest that TEs form a significantly higher percentage of coding sequences than represented in gene annotation databases and these TE derived sequences are epigenetically regulated in accordance with their expression in the two cell types.

## Introduction

A substantial fraction of the eukaryotic genome is comprised of transposable elements (TEs). In the human genome, more than half of the euchromatic sequence can be attributed to TEs. Despite their profusion, TEs have historically been regarded as *selfish* genomic parasites or *junk DNA* that have little to no contribution in the functioning of the human genome [1,2]. The aforementioned studies argued that TEs can maintain and proliferate in the host genome solely because of their ability to out-compete the host genome in terms of their replication ability, and are therefore not constrained to have a functional contribution necessary for selection. These studies shaped the paradigm for research in the field by discouraging the search for the functional relevance of TE sequences in the human genome.

Over the last several years, scientists discovered gene features such as promoters, UTR’s and exons derived from TE insertions that regulate transcription [3,4]. Exonization is a process whereby genes acquire new exons from non-protein-coding DNA sequences, often times TEs due to the presence of internal splice site-like structures within their sequences [5]. A classic example is the *agouti* locus in mice, where its expression results in a yellow coat color, obesity, diabetes, and tumor susceptibility [6]. The transcription of the *agouti* gene is influenced by a cryptic promoter originating from an intracisternal particle (IAP) element, a member of the LTR-Retrotransposon family, which generates a transcript that reads through the *agouti* gene. The number of transcripts originating from the IAP element depends on its methylation state where a de-methylated IAP element is capable of generating transcripts resulting in an increase in the expression of the *agouti* gene.

Indeed there are several instances where TEs have been implicated in functionally contributing to the human genome [7]. These contributions include TEs providing regulatory and coding sequences such as transcription factor binding sites, promoters, exons, and enhancers [8–12]. As is the case with the Agouti locus described above, TE derived gene features are often epigenetically regulated to cater to the functional needs of the cell type they are in. We have recently shown that TEs donate several hundred canonical promoters and transcription start sites to the human genome that are epigenetically modified in such a way as to facilitate cell type specific expression [13,14]. Similarly, we have also demonstrated that TEs donate thousands of unique enhancers that are modified by epigenetic histone modifications and are functionally relevant in driving cell type specific gene expression [14,15].

These studied provided the motivation to explore the global contribution of TE derived gene features in various human cell types and their potential role in gene regulation. The availability of RNA-sequencing (RNA-Seq) and epigenetic data from various human cell lines in the Encyclopedia of DNA Elements (ENCODE) project provides an unprecedented survey of the landscape of transcription genome-wide and, in a limited way, catalogues transcripts initiated from repetitive elements in a cell line specific manner [16]. However, there is a paucity of information regarding epigenetic regulation of TE-derived transcripts. To that end, we used the RNA-seq data as well as the epigenetic data provided by the ENCODE project in two human hematopoietic cell lines (GM12878 and K562)in order to ascertain the interplay between the exonization of TEs into coding sequences and epigenetic regulation of transcription on a genome-wide scale. GM12878 is a lymphoblastoid cell line derived from a female donor of northern and western European descent whereas K562 is a cancer cell line from a female patient suffering from Chronic Myelogenous Leukemia (CML). In each cell line, we found several thousand instances of TEs donating coding sequences to human genes and that TE exonization is significantly greater in the reference guided transcriptome assembly (Refseq [17] and Gencode [18]) than an assembly without the aid of a reference transcriptome. In addition, integration of histone modifications chromatin immunoprecipitation sequencing (ChIP-Seq) data, cap analysis of gene expression (CAGE) data and DNAseI hypersensitivity site (DHS) data allowed us to postulate that TEs contribute a substantial fraction of human coding sequences that are epigenetically regulated in accordance with the gene expression in the two cell types.

## Materials and Methods

### Datasets

RNA-seq data from GM12878 and K562 cell lines generated by Caltech were downloaded from the University of California Santa Cruz (UCSC) genome browser’s ENCODE downloads section and is also available in the NCBI GEO repository under accession #GSE23316. In each cell line, two replicate libraries of (75bpx2) paired-end reads were used for the analysis.

ChIP-seq data in GM12878 and K562 cell lines for four histone modifications (H3K4me3, H3K9ac, H3K27ac, H3K36me3) commonly found at transcription start sites and exons, were obtained as aligned tag files generated by the Broad institute and downloaded from the ENCODE section of the UCSC genome browser [19,20]. The data is also available in the NCBI GEO repository under accession #GSE29611.

Open chromatin data generated by Duke University under the ENCODE project using DHS mapping were also obtained as ‘Peaks’ file from the UCSC genome browser and is also available in the NCBI GEO repository under accession #GSE32970.

CAGE data generated by the RIKEN group for ENCODE cell lines, were also obtained from the UCSC genome browser as “CAGE Clusters” of loci deemed significantly enriched for CAGE tags against a background tag distribution. The raw data is also available in the NCBI GEO repository under accession #GSE34448.

The aforementioned datasets were co-located with transposable element (TE) annotation using custom Perl scripts as described by Repeat Masker version 3.2.7. The criteria for delineating TE derived RNA-seq exons were taken as any overlap between an exon and a TE with respect to genomic coordinates. Similarly, all other datasets (Histone modifications, DHS, CAGE) were co-located using genomic overlap between the TE annotation and the respective tag/peak/cluster densities.

### RNA-seq analysis

Raw paired-end sequence reads of length 75bpx2 with an insert size of 200bp were mapped individually to the reference human genome (hg19) using *Bowtie* (version 0.12.7) allowing at most 2 mismatches and multiple alignments for a read pair [21]. The *TopHat* (Cahnge oveer all in article) program was used with default parameters to ascertain splice junctions in the mapped reads [22]. *Cufflinks* (version 1.0.3) was used to assemble transcripts and estimate their abundance given a reference transcriptome specified by the Refseq and Gencode (version 7) gene models [18, 23,24]. As shown in (Figure S1), we used *Bowtie* to map the RNA-Seq reads to the hg19 genome, and then used *TopHat* to detect splice junctions and *Cufflinks* to assemble the transcriptome using two different techniques in order to determine the extent of TEs incorporation. First we built the transcriptome using reference annotations from Refseq [17] and Gencode [18] to ‘guide’ the assembly of RNA-seq reads. We also built the transcriptome without the aid of a reference gene model to yield a ‘transcript-reference-free’ characterization of RNA-seq reads. We co-located TE-annotations with RNA-seq data resulting from each assembly process and discovered that the fraction of TE-derived transcripts and exons is several folds higher in non-reference guided assembly. We further evaluated these data for functional signatures such as epigenetic histone modifications present at promoters and exons, cap analysis of gene expression (CAGE) data which identifies transcript start sites (TSS), and DNAseI hypersensitive sites (DHSs) which locate actively transcribing regions as characterized by open chromatin.

### Statistical analyses

A Student’s t-test-was used to compare gene expression transcripts from the non-reference guided assembly between GM12878 and K562 cell lines. False discovery rate (FDR) adjusted p-values (q-values) were used to control for multiple testing and to determine significance at q-value<0. 05. Gene Ontology and functional enrichment of differentially expressed genes were performed using the DAVID Bioinformatics Resources v6. 7 [25,26]. Pearson’s correlation (r) was also used to ascertain the relationship between various trends. The significance of a trend was determined by approximating the sampling distribution of *r* to the Student’s t-distribution using the formula:

1. $$t=r\sqrt{\frac{n-2}{1-r^{2}}}$$

## Results and Discussion

### TE-derived human gene transcripts

We obtained RNA-seq data generated by Caltech for the ENCODE project in GM12878 and K562 cell lines. These data were processed using the *Bowtie/TopHat/Cufflinks* pipeline as described in the Materials and Methods section. The reads were first mapped to the reference human genome (hg19) using *Bowtie* and the *TopHat* program was used to map splice junctions [21,22]. The third and final step in this pipeline is quantification by *Cufflinks* which assembles the transcripts and estimates their abundance [23,24,27]. Normalized transcript abundance is used as a measure of expression of the respective genes. The assembly and annotation of RNA-seq transcripts by *Cufflinks* can be aided by providing a reference transcriptome such as Refseq to assign the mapped reads and splice junctions to known transcripts. This process generally results in a higher number of transcripts discovered but is biased towards the transcripts that exist in the reference annotation. *Cufflinks* can also build the transcriptome without the use of reference annotation which can be used to detect a greater number of novel transcripts that do not exist in gene model databases.

**Figure 1:**
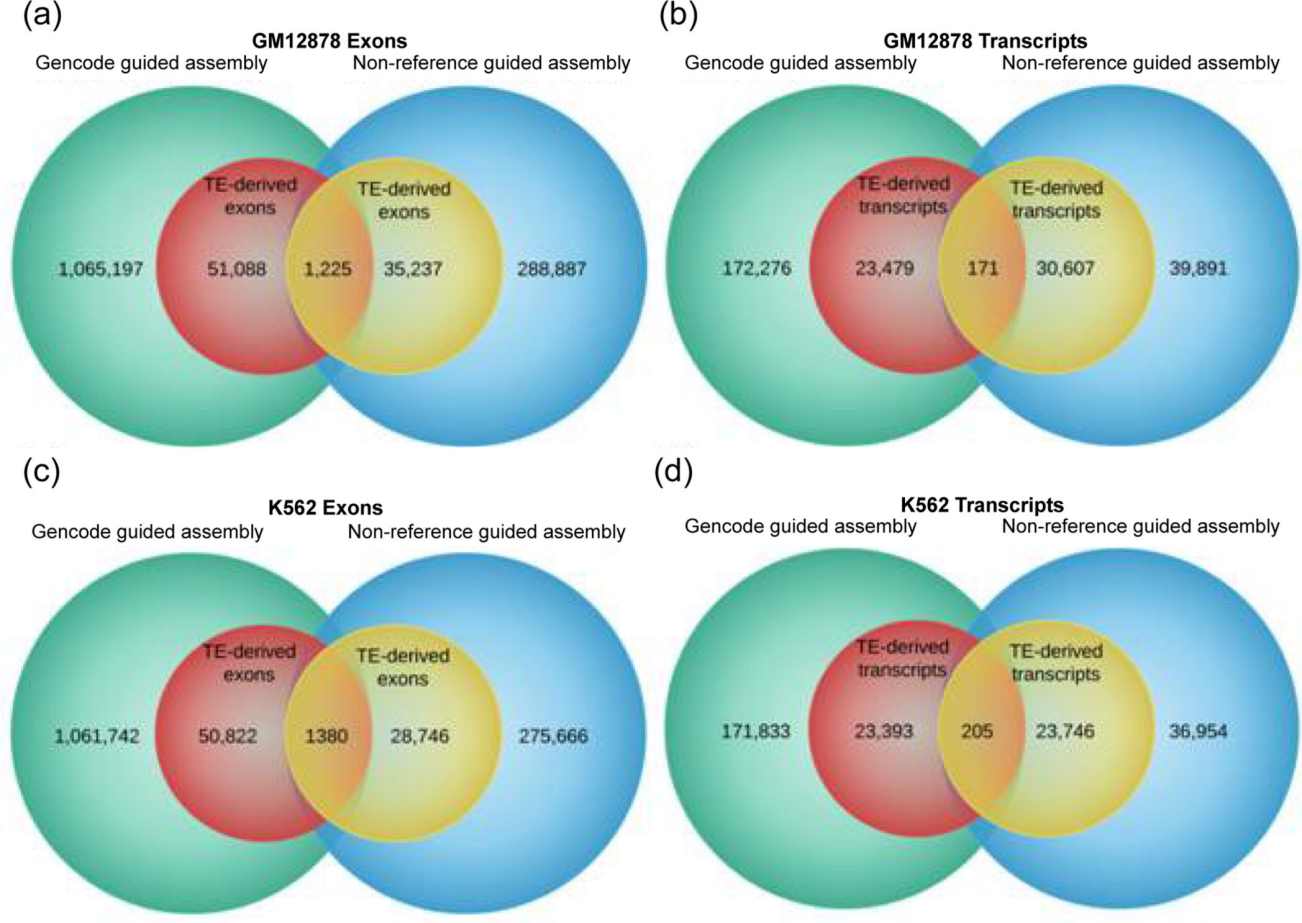
Comparison of Gencode guided versus non-reference guided assembly of transcripts and exons.The larger circles represent all transcripts and exons whereas the smaller circles represent TE-derived transcripts and exons (a) GM12878 exons (b) GM12878 transcripts (c) K562 exons (d) K562 transcripts.

**Figure 2:**
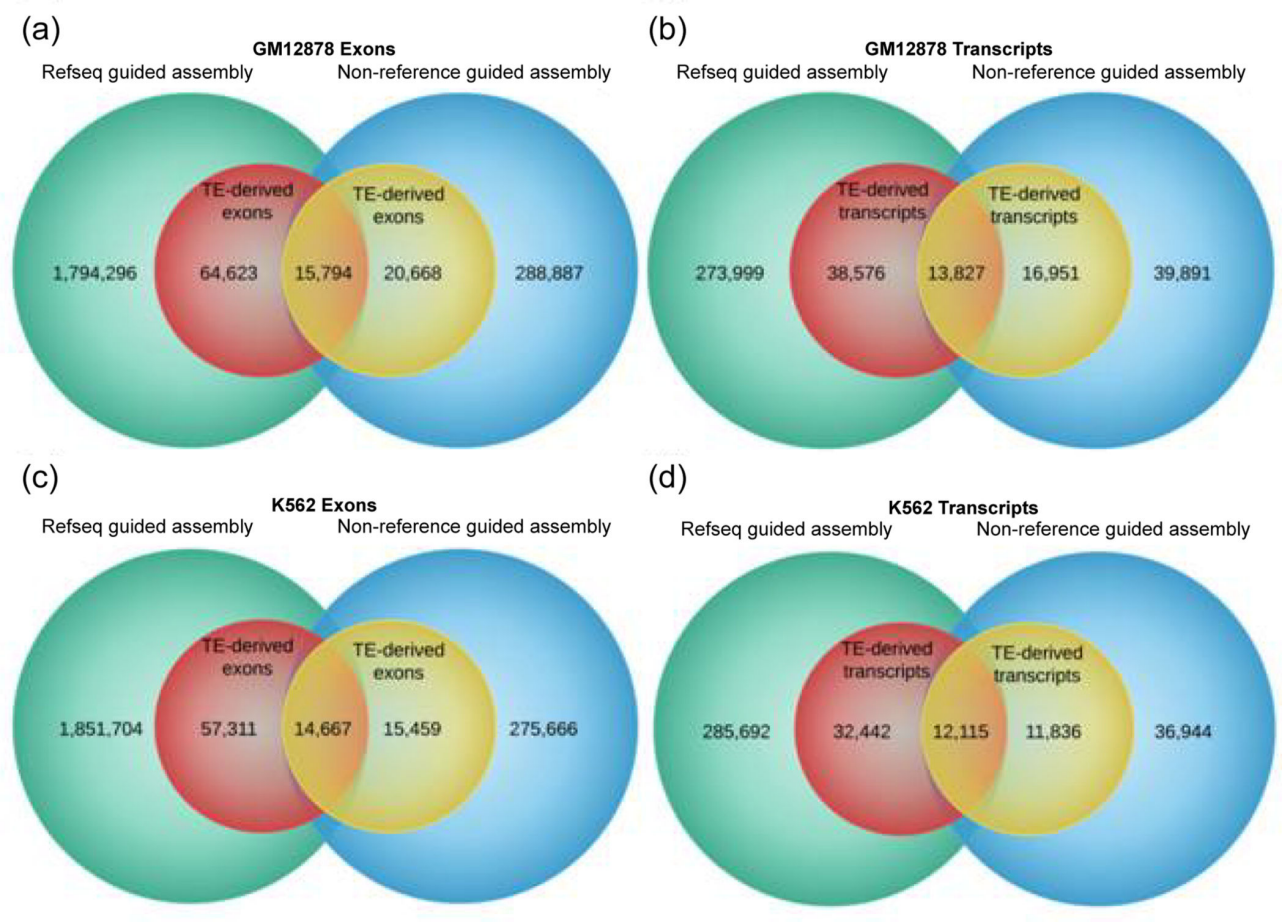
Comparison of Refseq guided *vs* non-reference guided assembly of transcripts and exons.The larger circles represent all transcripts and exons whereas the smaller circles represent TE-derived transcripts and exons (a) GM12878 exons (b) GM12878 transcripts (c) K562 exons (d) K562 transcripts.

**Figure 3:**
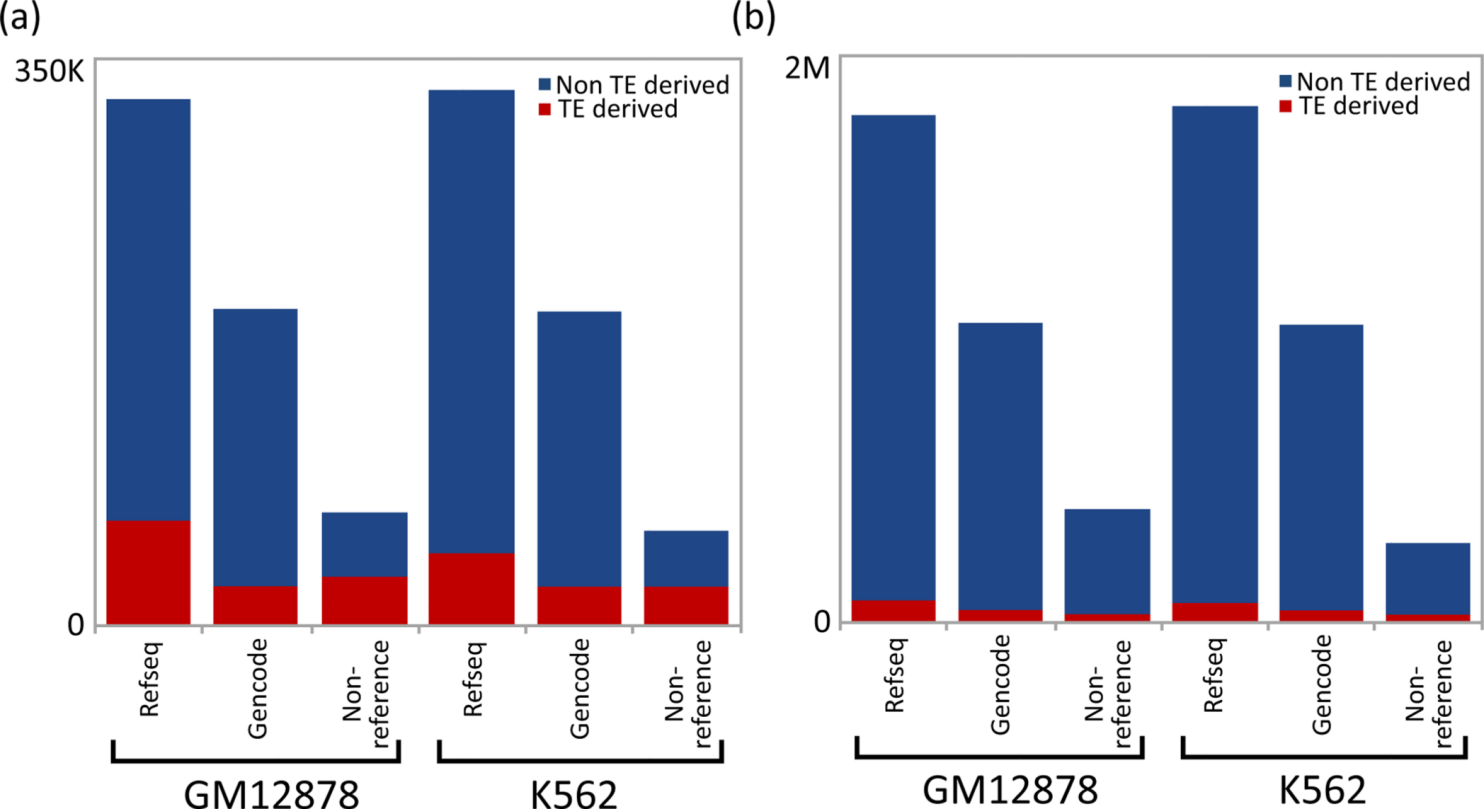
TE-derived (a) transcripts and (b) exons Illustrated as a fraction of total.

We ran Cufflinks in three different ways: without the aid of a reference transcriptome, using Refseq as reference annotation, and using Gencode as reference. This process resulted in vastly different results as illustrated in Figure 1 and Figure 2. In both cell lines, the use of a reference transcriptome during transcript assembly by Cufflinks resulted in the discovery of a considerably larger number of transcripts and exons. Transcriptome assembly using Refseq yielded the highest number of transcripts and exons, with (GM12878:1.87M and K562:1.92M) exons, and (GM12878:326K and K562:330K) transcripts. This was followed by a Gencode guided assembly which detected (GM12878:1.11M and K562:1.11M) exons and (GM12878:195K and K562:195K) transcripts in the two cell lines. On the other hand, non-reference guided assembly was able to discover only (GM12878:325K and K562:305K) exons and (GM12878:70K and K562:60K) transcripts in the two cell lines. Compared to non-reference guided assembly, these figures represent approximately 3X and 6X increase in the number of transcripts and exons discovered by Gencode and Refseq guided assembly, respectively (Figure 3 and Table 1).

To ascertain the contribution of transposable elements to human gene coding sequences, we co-located the transcripts and exons from each of the three assemblies with TE annotation as provided by Repeat Masker [28]. The term exonization in the context of TEs is described as the process by which a TE sequence is adapted to serve as an exon by the host genome. A transcript or exon is considered ‘TE-derived’ if any part of its coding sequence overlaps with TE annotation. The fraction of TE-derived coding sequences varies widely between the three transcriptome assemblies. As such, the non-guided assembly yields the highest proportion of TE-derived genes compared to Gencode and Refseq guided assemblies (Figure 3). These results indicate that TE-derived human gene coding sequences are vastly under-represented in the reference transcriptome annotations.

Having recognized the substantial under-representation in the fraction of TE-derived exons, we sought to determine the number of these coding sequences that are common between the reference and non-reference guided assemblies. To that end, we found all transcripts or exons from different builds that have common start and end positions in the genome. In order to compensate for possible imprecision in the prediction of transcripts and exon coordinates, we allowed a 10bp wiggle room in the comparison of start and end sites of transcripts and exons from different transcriptome assemblies. We found that a very small number of TE-derived transcripts and exons are in fact common between the reference and non-reference guided assemblies (Figures 1 and 2). Since the total number of transcripts and exons discovered by the non-reference guided assembly is considerably smaller than that of reference guided assembly, it follows that a proportionally small number of TE-derived exons are in fact represented in this dataset. In other words, the total number of TEs-derived exons detected by the non-reference guided assembly may only represent a small percentage of the actual number of TEs involved presumably in serving the coding needs of the human genome. As such, these results offer an unbiased estimate of the percentage of human coding sequences derived from TEs and indicate that the contribution in absolute terms may be significantly higher. Thus, these results reveal a significantly higher level of TE contribution to human exons than previously thought.

### TE-derived first exons

**Figure 4:**
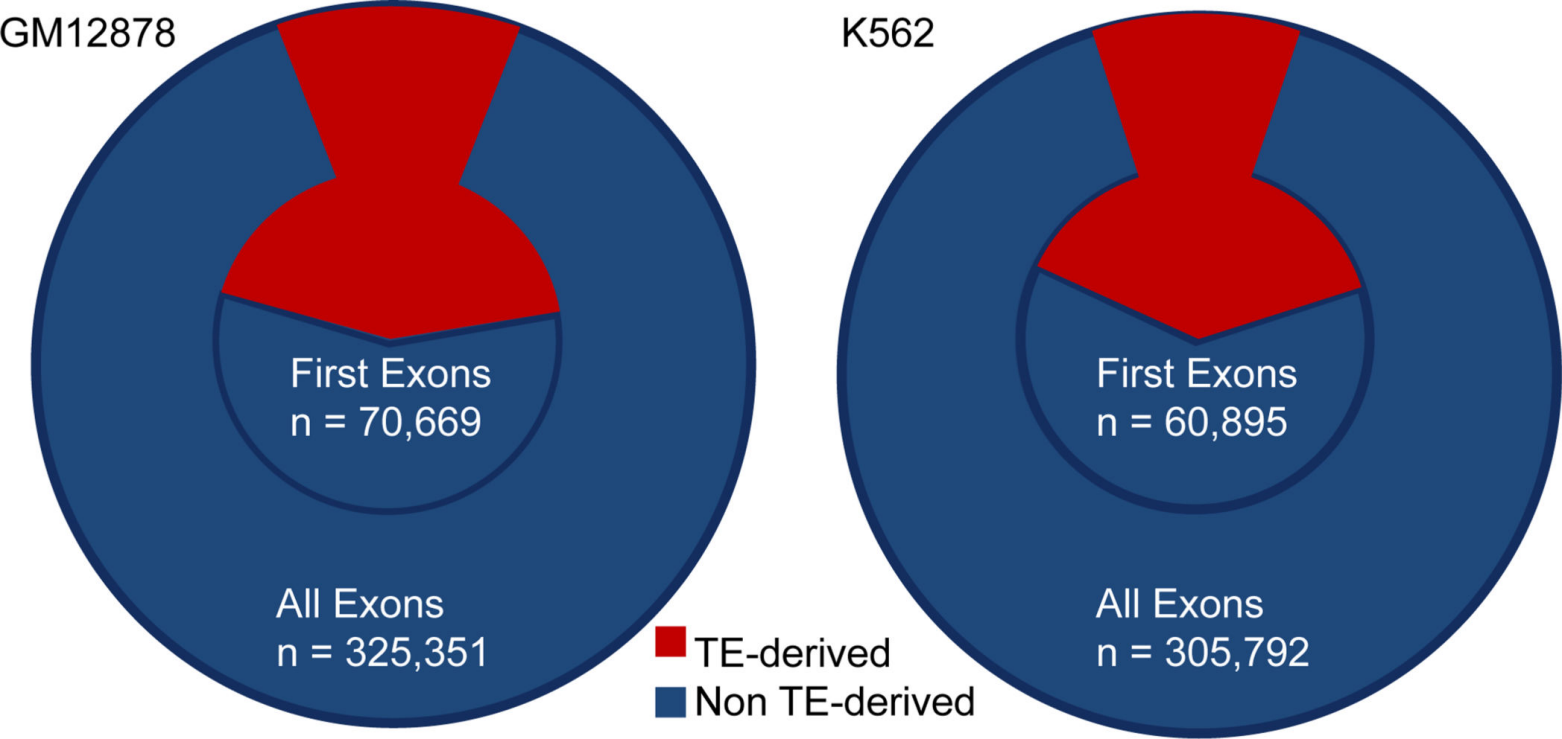
Fraction of TE-derived exons in all exons *vs* first exons. TE-derived exons form a signifcantly higher proportion of first exons compared to all exons (a) larger circle represents all exons while the smaller circle represents first exons and (b) TE-derived exons expressed as a percentage of total exons.

We evaluated the contribution of TEs in donating various gene segments including TSS (transcription start site) and first exons and compared it to subsequent exons. We report here that TEs are significantly over-represented in donating TSS and first exons as opposed to later exons (Figure 4 and Table 2). In the GM 12878 cell line (36,464/325,351) or 11.2% of all exons are derived from TEs, whereas (30,778/70,669) or 43.5% of first exons appear to be derived from TEs. Similarly, in the K562 cell line (30,126/305,792) or 9.9% of all exons are donated by TEs while (23,951/60,895) or 39.3% of first exons are TE-derived. Furthermore, we found 1291 transcripts (supplemental Table S1) mapped to 69 genes (Table 3) which are differentially expressed (q-value<0.05) between the GM12878 and K562 cell lines and have TEs inserted into the first exon of the genes. These genes primarily have molecular functions in the immunoglobulin receptor family class and enrich for the Gene Ontology immune response biological process (FDR<0.1). These data seem to support the model illustrated by the *agouti* gene in mouse where an IAP element (LTR Retrotransposon) residing upstream of the *agouti* locus generates a transcript that reads through the *agouti* gene [29]. In the process, the IAP element donates a transcription start site and is primed to serve as the first exon for the *agouti* gene. Our data suggest that this model is a common route through which TEs donate an alternative TSS and the first exon that can affect the transcription of nearby genes.

### Age and relative contribution of TE families

**Figure 5:**
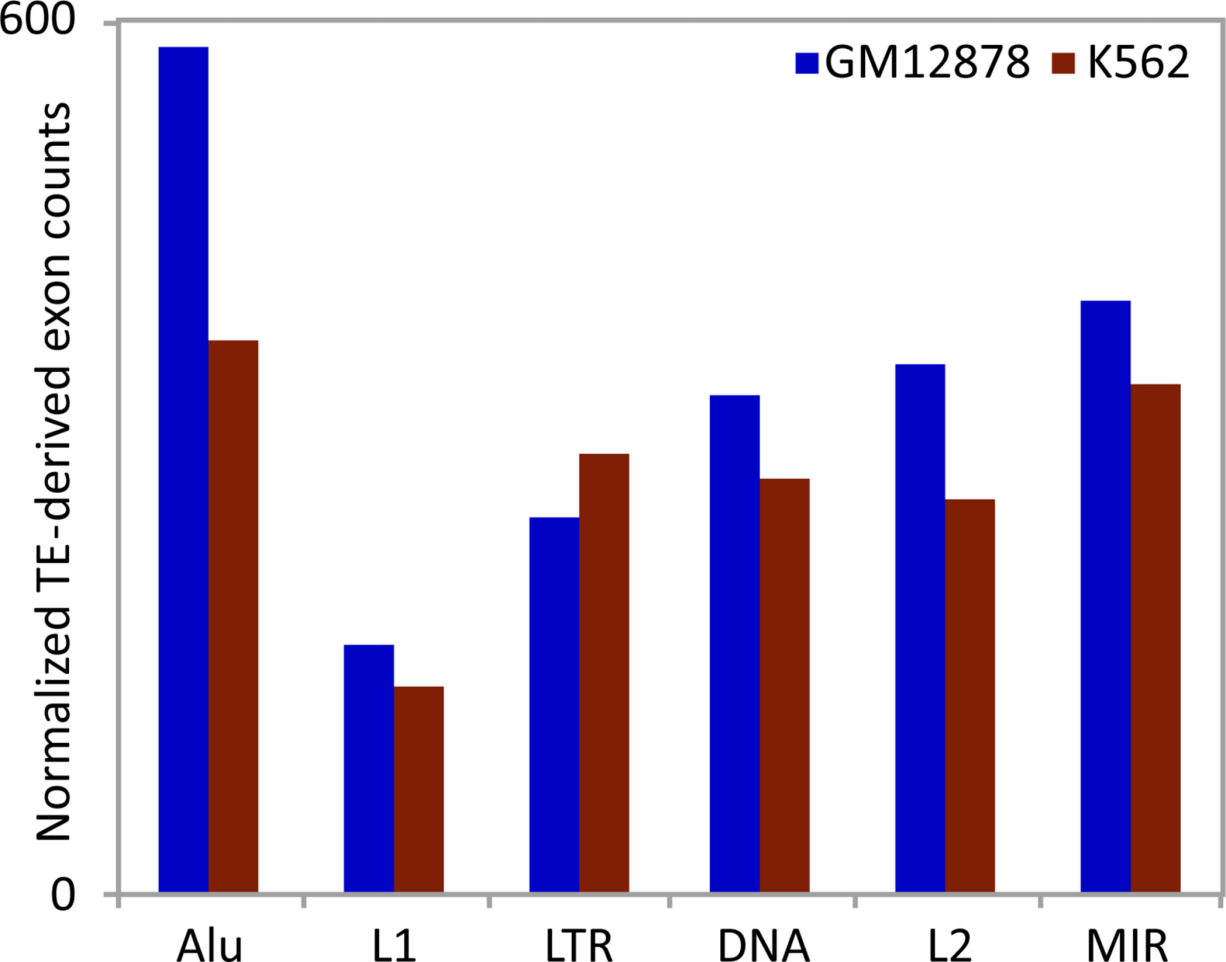
Relative contribution of TE-families in donating coding sequence to human gene transcripts. Absolute counts normalized by genomic background abundance for each family.

All major families of TEs are represented in contributing transcripts in the two cell types studied. We evaluated the relative contribution of various TE families in donating coding sequences in the RNA-seq transcripts by normalizing the number of transcripts attributed to a TE family with the background genomic abundance of the family. Different families of TEs have significantly (χ^2^ test p-value<0. 001) different contributions in providing coding sequences as illustrated in Figure 5.

We determined the relative age of each TE family by using their average divergence (millidiv) from a consensus ancestral sequence. Using this approach, we classified the TE families in the following order according to age, from youngest to the oldest: Alu, L1, LTR-Retrotransposons, DNA-transposons, L2 and MIR. We observed that the relative contribution of each TE family increases with increasing age of the family with the exception of Alu elements. Intuitively, older TE families have had a longer period of time for individual elements to be adopted by the genome and thus have relatively higher contribution compared to the younger families. Our observations are consistent with this phenomenon as older TE families are significantly more enriched in gene features. On the other hand, the youngest family of TEs i.e., Alu elements do not follow this paradigm and are highly enriched in donating coding sequences. It has previously been shown that Alu elements are enriched in gene rich regions which can be attributed to their over-representation in donating coding sequences [30].

### Epigenetic regulation of TE-derived genes

To strengthen the functional relevance of TE-derived exons, we evaluated the various epigenetic markers that are known to mark active exons. To that end, we downloaded histone modification data for four histone modifications (H3K4me3, H3K9ac, H3K27ac, and H3K36me3), DHS data as well as CAGE data. These four histone modifications have been shown to mark actively transcribing TSSs and exons [31–33]. Similarly, CAGE data is an established technique to determine the TSSs for actively transcribing genes [34]. Finally, DHSs are used to identify open chromatin and are shown to be correlated with activation of gene expression [35,36].

**Figure 6:**
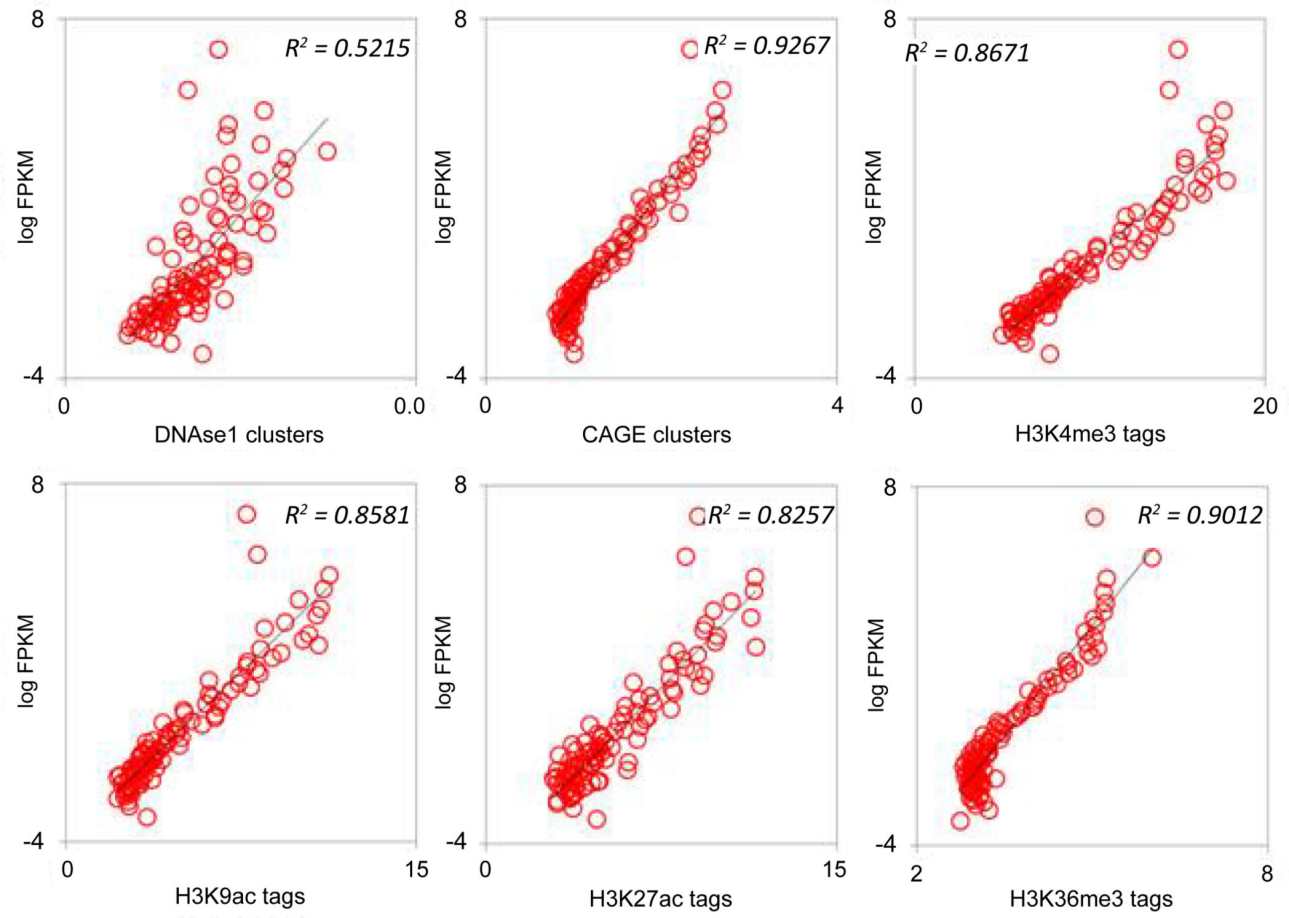
FPKM values for all TE-derived exons were sorted and binned in 100 equal sized bins and plotted against DHS clusters, CAGE clusters, and various histone modifications that mark promoters, TSS and exons in the GM12878 cell line.

**Figure 7:**
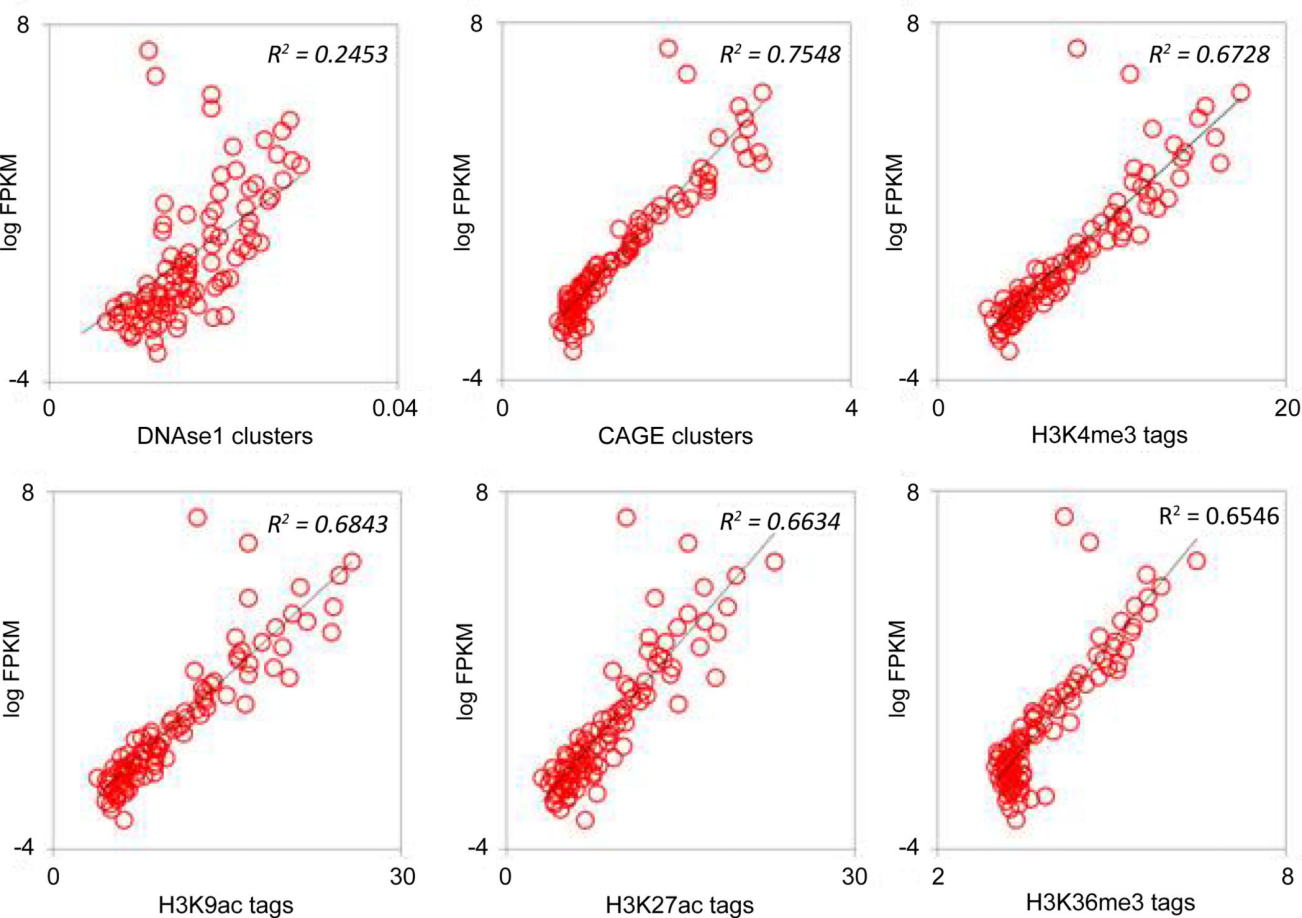
FPKM values for all TE-derived exons were sorted and binned in 100 equal sized bins and plotted against DHS clusters, CAGE clusters, and various histone modifications that mark promoters, TSS and exons in the K562 cell line.

These data were mapped to the TE-derived exons using their genomic loci. We divided the TE-derived exons into 100 bins according to their expression in the two cell lines and plotted their average epigenetic modification against their expression (Figures 6 and 7). In both cell lines, the expression of TE-derived exons is strongly positively correlated with the histone modifications, DHSs, and CAGE clusters. These epigenetic indicators of gene expression demonstrate the functional significance of TE-derived exons in presumably regulating gene expression in their respective cell types. Our data further suggests that transposable elements that donate coding sequences to human genes have been epigenetically modified in such a way as to facilitate their role in driving gene expression.

## Conclusions

Our data suggests that TEs have a substantial contribution in donating coding sequences to human genes. The level of contribution is made apparent by transcriptome assembly without the aid of a reference transcriptome and far exceeds that provided by the reference transcriptome. TE-derived exons and transcripts are also epigenetically regulated in such a way as to potentially facilitate the establishment of cell type specific gene expression. Our results in no way discounts or diminishes the presumed importance of TEs in non-coding regions but we make an association between exonization of TEs and epigenetic regulation of transcription. Furthermore, our interpretation of the results mainly centered on the findings from the reference guided assembly of the transcripts sequence reads as we could leverage the gene model as annotation for associating with the epigenetic data. Thus, one can ascertain that there are potentially novel transcripts from the non-reference guided assembly that are presumably as important as the epigenetically regulated ones we detected. Further investigation into these potential transcript regulators is of interest and we are also considering computational ways to discern false positive and false negative detection of TE exonization using synthetic reads and/or spike-ins in the absence of the ground truth.

## Figures and Tables

**Table 1:** Percentage of TE-derived transcripts and exons as a function of total transcripts and exons in GM12878 and K562 cell lines, refer to Figure 3.

	Transcripts		Exons	
	GM12878	K562	GM12878	K562
Refseq	19.6	13.4	4.2	3.7
Gencode	12.1	12.1	4.6	4.7
Non-reference	43.5	39.3	11.2	9.8

**Table 2:** Percentage of TE-derived first exons as a fraction of all exons in GM12878 and K562 cell lines, refer to Figure 4.

		All exons	First exons
GM12878	TE derived	11.2	43.5
	Non TE derived	88.8	56.5
K562	TE derived	9.9	39.3
	Non TE derived	90.1	60.7

**Table 3:** Significantly differentially expressed genes (q-value<0.05) between GM12878 and K562 cell lines and have TE inserted into the first exon of the genes.

Representative transcript	Uni Gene Cluster	Gene name	Gene symbol
NM_003975	Hs.103527	SH2 domain containing 2A	SH2D2A
NM_001039477	Hs.10649	Chromosome 1 open reading frame 38	C1orf38
NM_001146310	Hs.107101	Chromosome 1 open reading frame 86	C1orf86
NM_019089	Hs.118727	Hairy and enhancer of split 2 (Drosophila)	HES2
NM_018420	Hs.125482	Solute carrier family 22, member 15	SLC22A15
NM_033312	Hs.127411	CDC14 cell division cycle 14 homolog A (S. cerevisiae)	CDC14A
NM_001765	Hs.132448	CD1c molecule	CD1C
NM_016074	Hs.13880	BolA homolog 1 (E. coli)	BOLA1
NM_001080471	Hs.142003	Platelet endothelial aggregation receptor 1	PEAR1
NM_001042747	Hs.1422	Gardner-Rasheed feline sarcoma viral (v-fgr) oncogene homolog	FGR
NM_001766	Hs.1799	CD1d molecule	CD1D
NM_004672	Hs.194694	Mitogen-activated protein kinase kinasekinase 6	MAP3K6
NM_148902	Hs.212680	Tumor necrosis factor receptor superfamily, member 18	TNFRSF18
NM_030812	Hs.2149	Actin-like 8	ACTL8
NM_003528	Hs.2178	Histone cluster 2, H2be	HIST2H2BE
NM_001166294	Hs.22587	Synovial sarcoma, X breakpoint 2 interacting protein	SSX2IP
NM_014215	Hs.248138	Insulin receptor-related receptor	INSRR
NM_173452	Hs.333383	Ficolin (collagen/fibrinogen domain containing) 3 (Hakata antigen)	FCN3
NM_006824	Hs.346868	EBNA1 binding protein 2	EBNA1BP2
NM_001007794	Hs.363572	Choline/ethanolamine phosphotransferase 1	CEPT1
NM_147192	Hs.375623	Diencephalon/mesencephalon homeobox 1	DMBX1
NM_002529	Hs.406293	Neurotrophic tyrosine kinase, receptor, type 1	NTRK1
NM_003517	Hs.408067	Histone cluster 2, H2ac	HIST2H2AC
NM_001193300	Hs.408846	Sema domain, immunoglobulin domain (Ig), transmembrane domain (TM) and short cytoplasmic domain, (semaphorin) 4A	SEMA4A
NM_052941	Hs.409925	Guanylate binding protein 4	GBP4
NM_015696	Hs.43728	Glutathione peroxidase 7	GPX7
NM_001143778	Hs.437379	ArfGAP with SH3 domain, ankyrin repeat and PH domain 3	ASAP3
NM_030764	Hs.437393	Fc receptor-like 2	FCRL2
NM_001033082	Hs.437922	V-mycmyelocytomatosis viral oncogene homolog 1, lung carcinoma derived (avian)	MYCL1
NM_198715	Hs.445000	Prostaglandin E receptor 3 (subtype EP3)	PTGER3
NM_003196	Hs.446354	Transcription elongation factor A (SII), 3	TCEA3
NM_005101	Hs.458485	ISG15 ubiquitin-like modifer	ISG15
NM_001195740	Hs.462033	Chromosome 1 open reading frame 93	C1orf93
NM_001040195	Hs.464438	Angiotensin II receptor-associated protein	AGTRAP
NM_001048195	Hs.469723	Regulator of chromosome condensation 1	RCC1
NM_024772	Hs.471243	Zinc finger, MYM-type 1	ZMYM1
NM_002959	Hs.485195	Sortilin 1	SORT1
NM_001199772	Hs.485246	Proteasome (prosome, macropain) subunit, alpha type, 5	PSMA5
NM_178454	Hs.485606	DNA-damage regulated autophagy modulator 2	DRAM2
NM_002744	Hs.496255	Protein kinase C, zeta	PRKCZ
NM_001143989	Hs.511849	Neuroblastoma breakpoint family, member 4	NBPF4
NM_003820	Hs.512898	Tumor necrosis factor receptor superfamily, member 14 (herpesvirus entry mediator)	TNFRSF14
NM_004000	Hs.514840	Chitinase 3-like 2	CHI3L2
NM_025008	Hs.516243	ADAMTS-like 4	ADAMTSL4
NM_001178062	Hs.516316	Sema domain, transmembrane domain (TM), and cytoplasmic domain, (semaphorin) 6C	SEMA6C
NM_021181	Hs.517265	SLAM family member 7	SLAMF7
NM_022873	Hs.523847	Interferon, alpha-inducible protein 6	IFI6
NM_152493	Hs.524248	Zinc finger protein 362	ZNF362
NM_000760	Hs.524517	Colony stimulating factor 3 receptor (granulocyte)	CSF3R
NM_001135585	Hs.530003	Solute carrier family 2 (facilitated glucose/fructose transporter), member 5	SLC2A5
NM_033504	Hs.534521	Transmembrane protein 54	TMEM54
NM_024901	Hs.557850	DENN/MADD domain containing 2D	DENND2D
NM_005529	Hs.562227	Heparansulfate proteoglycan 2	HSPG2
NM_001408	Hs.57652	Cadherin, EGF LAG seven-pass G-type receptor 2 (flamingo homolog, Drosophila)	CELSR2
NM_033467	Hs.591453	Membrane metallo-endopeptidase-like 1	MMEL1
NM_024980	Hs.632367	G protein-coupled receptor 157	GPR157
NM_002143	Hs.632391	Hippocalcin	HPCA
NM_014787	Hs.647643	DnaJ (Hsp40) homolog, subfamily C, member 6	DNAJC6
NM_052938	Hs.656112	Fc receptor-like 1	FCRL1
NM_152890	Hs.659516	Collagen, type XXIV, alpha 1	COL24A1
NM_175065	Hs.664173	Histone cluster 2, H2ab	HIST2H2AB
NM_207397	Hs.664836	CD164 sialomucin-like 2	CD164L2
NM_001114748	Hs.668654	Chromosome 1 open reading frame 70	C1orf70
NM_144701	Hs.677426	Interleukin 23 receptor	IL23R
NM_001013693	Hs.710255	Low density lipoprotein receptor class A domain containing 2	LDLRAD2
NM_001037675	Hs.714127	Neuroblastoma breakpoint family, member 1	NBPF1
NM_152498	Hs.729552	WD repeat domain 65	WDR65
NM_001127714	Hs.729693	Human immunodeficiency virus type I enhancer binding protein 3	HIVEP3
NM_001164722	Hs.77542	Platelet-activating factor receptor	PTAFR

## Abbreviations

RNA: Ribonucleic acid
RNA-Seq: RNA sequencing
TE: Transposable Element
ChIP-Seq: Chromatin Immunoprecipitation Sequencing
DNA: Deoxyribonucleic Acid
DHS: DNAseI Hypersensitivity Site
ENCODE: Encyclopedia of DNA Elements
LTR: Long Terminal Repeats
IAP: Intracisternal A Particle
TSS: Transcription Start Site
NCBI: National Center for Biotechnology Information
GEO: Gene Expression Omnibus
RIKEN: RikagakuKenkyūjo
UCSC: University of California-Santa Cruz
FDR: False Discovery Rate
